# Optimal Stubble Management Strategies of *Caragana tibetica* for Enhancing Stress Resistance and Vegetation Restoration

**DOI:** 10.3390/plants14243867

**Published:** 2025-12-18

**Authors:** Xiaoman Yuan, Yong Gao, Yumei Liang

**Affiliations:** College of Desert Control Science and Engineering, Inner Mongolia Agricultural University, Hohhot 010018, China; yxm2002202308@163.com (X.Y.); liang490002878@163.com (Y.L.)

**Keywords:** desert steppe, *Caragana tibetica*, stubble intensity, stress resistance, physiological response, abiotic stress, grazing, ROS

## Abstract

*Caragana tibetica* Kom. is a key constructive species in desert steppe and desert transition zones. Long-term enclosure has led to population decline and even mortality of *C. tibetica*, while populations outside enclosures grow well. However, the biological mechanisms underlying the continued growth of *C. tibetica* under grazing remain unclear. Therefore, this study aimed to clarify the effects of stubble management on the photosynthetic physiology and antioxidant characteristics of *C. tibetica*, and to determine the optimal stubble intensity. Plants were subjected to five stubble gradients (0%, 25%, 50%, 75%, 100%). The results showed that stubble treatments caused significant changes in both photosynthetic and antioxidant traits. Interestingly, the correlations between photosynthetic and antioxidant characteristics varied with the growth season: they were positively correlated in the early growth season, but negatively correlated in the middle and late stages. Using a generalized algorithmic model, we found that stubble intensities ranging from 0.5% to 38.7% enhanced the stress resistance of *C. tibetica*, with 21.6% being the optimal intensity. This study demonstrates that moderate stubble management promotes the stress resistance of *C. tibetica*, providing important theoretical and scientific support for vegetation restoration and ecological construction in desert steppes.

## 1. Introduction

*Caragana tibetica* Kom. (*C. tibetica*) is an arid shrub native to East Asia that provides local farmers with supplementary livestock forage (leaves and flowers) and fuel resources (branches), and assists in soil and water conservation. It is widely used for the restoration of degraded land. Due to its biological characteristics, *C. tibetica* exhibits an umbrella-shaped structure and, with its well-developed root system and hardened spines, possesses strong drought resistance. It is a dominant species in the ecotone between desert steppe and desert [1,2]. However, with the increasing intensity of human activities and climate change, the population of *C. tibetica* will face destruction. Studies have shown that climate change is expected to significantly reduce the suitable habitat area for *C. tibetica*, potentially shrinking it by 60% to 80% [3]. Fencing can improve plant productivity and promote the recovery of plant communities [4]. In our team’s previous studies on the conservation of rare and endangered plants, it was found that initial fencing allows *C. tibetica* to gradually restore its ecological functions. However, with long-term fencing, *C. tibetica* exhibits growth decline and even mortality, resulting in reduced population stability. In contrast, compared to fenced areas, *C. tibetica* in free grazing lands demonstrates overall vigorous growth and higher population stability. This study hypothesizes that livestock grazing in free-grazing lands helps reduce apical dominance and adjusts source-sink allocation, thereby enhancing population diversity and stability. Although *C. tibetica* continues to grow after grazing, its biological characteristics of compensatory growth in response to livestock foraging remain unclear.

Stubble cutting is a common plant management practice that can inhibit plant senescence, enhance plant productivity, and improve growth status [5,6]. Photosynthesis forms the basis of plant growth and is highly sensitive to external environmental changes such as solar radiation, atmospheric temperature, and humidity [7]. Moderate stubble cutting can increase the net photosynthetic rate, accelerate aboveground biomass production, and improve post-cutting recovery capacity. Studies have shown that stubble cutting can alleviate the inhibition caused by apical dominance. Compared to uncut plants, those subjected to stubble cutting exhibit enhanced photosynthetic capacity and higher productivity [8,9]. Additionally, under environmental stress, plants reduce their ability to eliminate reactive oxygen species (ROS), leading to ROS accumulation and exacerbating cellular membrane damage [10]. Malondialdehyde (MDA) is a key indicator of the extent of damage to plant cell membranes [11,12]. When plants are stressed, MDA content rises; plants respond by increasing soluble protein (SP) to regulate osmotic pressure and raising soluble sugar (SS) levels to accelerate recovery, thereby enhancing their survival under adversity [13]. At the same time, the plant antioxidant system undergoes coordinated changes under stress [14]. Research indicates that the removal of apical buds can strengthen the activity of antioxidant enzymes, such as superoxide dismutase (SOD), catalase (CAT), and peroxidase (POD) [15]. The antioxidant system mitigates cell damage by increasing the activity of ROS-scavenging enzymes to reduce ROS accumulation [16,17]. Based on this, the present study uses gradient stubble cutting to replace livestock grazing and clarifies the relationship between stubble intensity and the physiological characteristics of *C. tibetica*. It aims to explore the optimal stubble intensity for *C. tibetica*, addressing the balance between population conservation and practical production. The ultimate goal is to provide theoretical support for establishing efficient, stable, and sustainable management systems for natural reserves.

## 2. Results

### 2.1. Analysis of Photosynthetic Physiological Characteristics of Caragana tibetica

**Figure 1:**
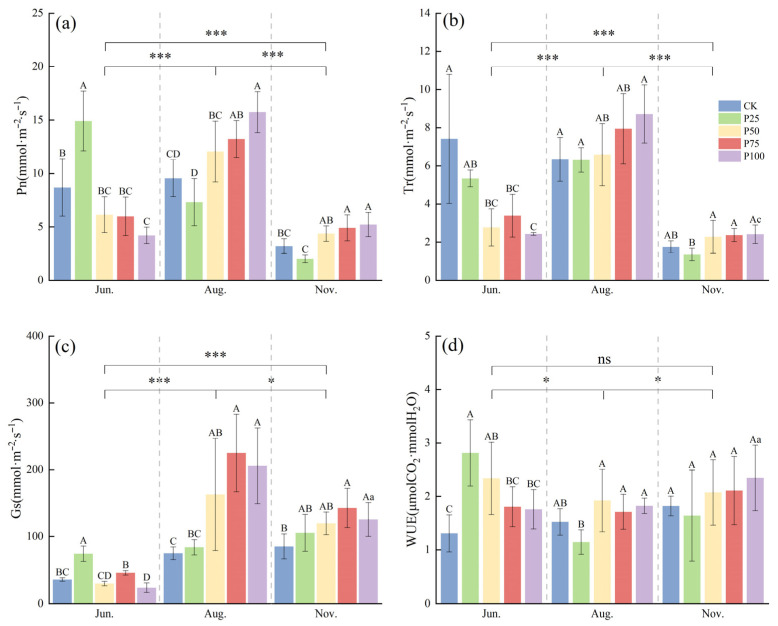
Effects of cutting on photosynthetic physiological characteristics of *Caragana tibetica*. (**a**) Net photosynthetic rate (Pn); (**b**) Transpiration rate (Tr); (**c**) Stomatal conductance (Gs); (**d**) Water use efficiency (WUE). Data are expressed as SE ± SD. Different uppercase and lowercase letters indicate significant differences between treatments based on one-way ANOVA (*p* ≤ 0.05); * indicates significant differences between months determined by LSD test with significance levels: *p* ≤ 0.05 (*), *p* ≤ 0.001 (***), *p* > 0.05 (ns). Statistical analysis was performed using one-way ANOVA followed by LSD post hoc test for monthly comparisons, and variability across the entire season was assessed. *n* = 5.

To investigate the effects of different cutting intensities on the photosynthetic physiological characteristics of *C. tibetica*, this study analyzed variations in net photosynthetic rate (Pn) and transpiration rate (Tr) to reveal differences among treatments. As shown in Figure 1, both Pn and Tr were significantly higher in June and August compared to November (*p* ≤ 0.05), with all treatments reaching their maximum values in August. Specifically, in June, the Pn of the P25 treatment was significantly higher than that of the other treatments (*p* ≤ 0.05), and the overall trend showed an initial increase followed by a decrease. In August, the Pn peaked, with P100 and P75 treatments significantly higher than CK (*p* ≤ 0.05), displaying an overall trend of initial decrease followed by an increase. In November, the Pn declined across all treatments, but P100 and P75 remained significantly higher than CK (*p* ≤ 0.05). For the Tr, the P50, P75, and P100 treatments were significantly lower than CK in June (*p* ≤ 0.05), showing a gradual decreasing trend. In August and November, there were no significant differences between the treatments and CK (*p* > 0.05), and the overall trend was a gradual decline.

To understand the effects of different treatments on water resource utilization, this study analyzed the changes in stomatal conductance (Gs) and water use efficiency (WUE). Gs in August and November was significantly higher than in June (*p* ≤ 0.05). In August, Gs reached its peak, with the P50, P75, and P100 treatments significantly higher than CK (*p* ≤ 0.05), indicating enhanced stomatal regulation capacity. In June and November, Gs was relatively low, and significant differences were observed among treatments, suggesting that stomatal opening and closing were notably influenced by cutting intensity and seasonal factors. WUE exhibited different trends across the months. In June, WUE for the P25 and P50 treatments was significantly higher than that of CK (*p* ≤ 0.05), indicating that cutting treatments improved WUE. Differences among treatments diminished in August, while WUE overall increased again in November. The results demonstrate that moderate cutting enhances stomatal regulation and WUE, enabling *C. tibetica* to maintain relatively high photosynthetic efficiency during dry periods and highlighting its adaptability to water stress.

### 2.2. Analysis of Antioxidant Characteristics of Caragana tibetica

**Figure 2:**
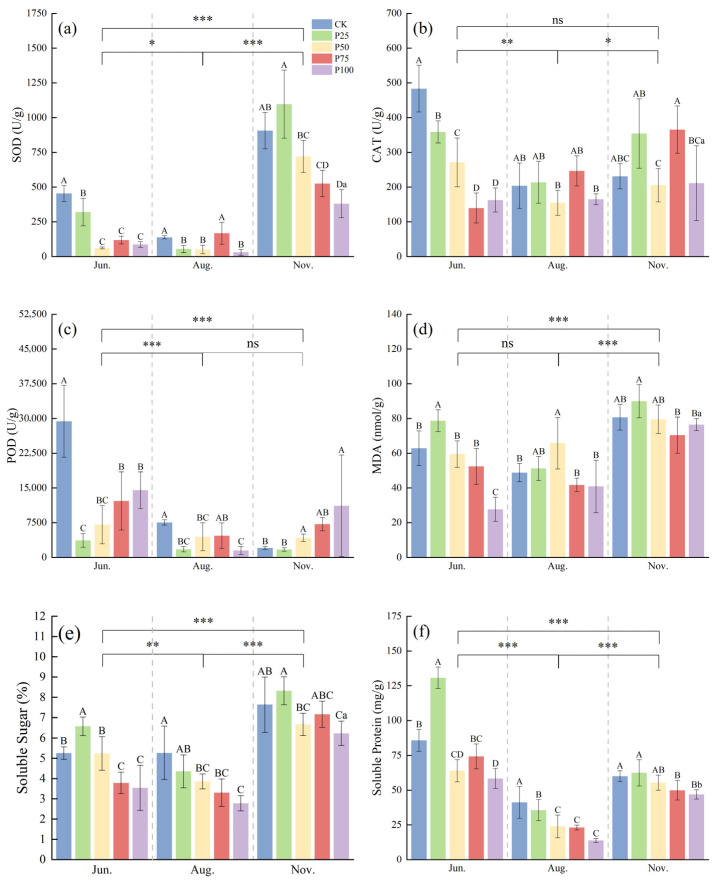
Effects of cutting on antioxidant physiological characteristics of *Caragana tibetica*. (**a**) Superoxide dismutase (SOD); (**b**) Catalase (CAT); (**c**) Peroxidase (POD); (**d**) Malondialdehyde (MDA); (**e**) Soluble sugar (SS); (**f**) Soluble protein (SP). Data are expressed as SE ± SD. Different uppercase and lowercase letters indicate significant differences between treatments based on one-way ANOVA (*p* ≤ 0.05); * indicates significant differences between months determined by LSD test with significance levels: *p* ≤ 0.05 (*), *p* ≤ 0.01 (**), *p* ≤ 0.001 (***), *p* > 0.05 (ns). Statistical analysis was performed using one-way ANOVA followed by LSD post hoc test for monthly comparisons, and variability across the entire season was assessed. *n* = 5.

According to Figure 2, the activities of SOD, MDA, and SS in November were significantly higher than those in June and August (*p* ≤ 0.05), while the activities of CAT, POD, and SP in June were significantly higher than those in August and November (*p* ≤ 0.05). In June, the SOD, CAT, and POD activities of P25, P50, P75, and P100 were significantly lower than those of CK (*p* ≤ 0.05), and with increasing cutting intensity, the activities of SOD, CAT, and POD first decreased and then increased. With increasing cutting intensity, the contents of MDA, SS, and SP first increased and then decreased. The contents of MDA, SS, and SP in P25 were significantly higher than those in CK (*p* ≤ 0.05). In August, the SOD, CAT, and POD activities of P50 and P100 were significantly lower than those of CK (*p* ≤ 0.05), while the activities of SOD, CAT, and POD in P75 showed no significant difference compared with CK (*p* > 0.05). The MDA content of P50 was significantly higher than that of CK (*p* ≤ 0.05), and the SS and SP contents of P50, P75, and P100 were significantly lower than those of CK (*p* ≤ 0.05). As the cutting intensity increased, the activities of SOD, CAT, POD, and the content of MDA first decreased and then increased, while the contents of SS and SP showed a gradual decline. In November, the SOD activity of P25 was significantly higher than that of CK (*p* ≤ 0.05), the POD activity of P100 was significantly higher than that of CK (*p* ≤ 0.05), and the SS and SP contents of P100 were significantly lower than those of CK (*p* ≤ 0.05). For other indicators, no significant differences were observed between treatments and CK. In summary, moderate cutting treatments in different seasons help to enhance the antioxidant enzyme activities of *C. tibetica*, reduce the content of lipid peroxidation product MDA, and regulate the levels of soluble sugars and proteins, indicating that cutting has a notable seasonal regulatory effect on its stress resistance.

### 2.3. Correlation Analysis of Photosynthetic Physiological Characteristics, Antioxidant Enzyme Activities, and Soluble Substances in Caragana tibetica

**Figure 3:**
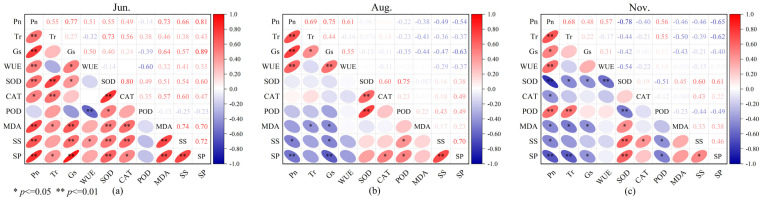
Correlation analysis of photosynthetic physiological characteristics, antioxidant enzyme activities, and soluble substances in *Caragana tibetica.* Pearson correlation of physiological indicators in June (**a**), August (**b**), and November (**c**). The color scale represents the range of correlation coefficients, with red indicating positive correlation and blue indicating negative correlation.

To explore the relationships between photosynthetic physiological characteristics and enzyme activities in *C. tibetica*, this study conducted a correlation analysis of parameters including photosynthetic physiological traits, antioxidant enzyme activities, and soluble substance contents. As shown in Figure 3, there were significant correlations among the various parameters. Specifically, in June, Pn was extremely significantly and positively correlated with Gs, SS, SP, and MDA (*p* ≤ 0.01, *R* = 0.77; *p* ≤ 0.01, *R* = 0.66; *p* ≤ 0.01, *R* = 0.81; *p* ≤ 0.01, *R* = 0.73, respectively). Tr showed an extremely significant positive correlation with SOD (*p* ≤ 0.01, *R* = 0.73). Gs was extremely significantly and positively correlated with SP and MDA (*p* ≤ 0.01, *R* = 0.89; *p* ≤ 0.01, *R* = 0.64). WUE was extremely significantly and negatively correlated with POD (*p* ≤ 0.01, *R* = −0.60). SS was extremely significantly and positively correlated with SP, CAT, and MDA (*p* ≤ 0.01, *R* = 0.72; *p* ≤ 0.01, *R* = 0.60; *p* ≤ 0.01, *R* = 0.74). SP was extremely significantly and positively correlated with MDA and SOD (*p* ≤ 0.01, *R* = 0.70; *p* ≤ 0.01, *R* = 0.60). CAT and SOD were extremely significantly and positively correlated (*p* ≤ 0.01, *R* = 0.80). In August, Pn was extremely significantly and positively correlated with Tr, Gs, and WUE (*p* ≤ 0.01, *R* = 0.69; *p* ≤ 0.01, *R* = 0.75; *p* ≤ 0.01, *R* = 0.61). Gs was extremely significantly and negatively correlated with SP (*p* ≤ 0.01, *R* = −0.63). SOD was extremely significantly and positively correlated with CAT and POD (*p* ≤ 0.01, *R* = 0.60; *p* ≤ 0.01, *R* = 0.75). SS was extremely significantly and positively correlated with SP (*p* ≤ 0.01, *R* = 0.70). In November, Pn was extremely significantly correlated with Tr, SOD, and SP (*p* ≤ 0.01, *R* = 0.68; *p* ≤ 0.01, *R* = −0.78; *p* ≤ 0.01, *R* = −0.65). Tr was extremely significantly and negatively correlated with SP (*p* ≤ 0.01, *R* = −0.62). SOD was extremely significantly and positively correlated with SS and SP (*p* ≤ 0.01, *R* = 0.60; *p* ≤ 0.01, *R* = 0.61).

These findings indicate that there are multiple types of correlations among different physiological indicators, which also exhibit seasonal variation. This intricate and overlapping correlation network suggests that the stress resistance of *C. tibetica* is the result of the coordinated regulation of various physiological mechanisms, providing a physiological basis for its adaptation to the arid environment.

### 2.4. Principal Component Analysis of Physiological Indicators and Their Comprehensive Performance in Caragana tibetica

**Figure 4:**
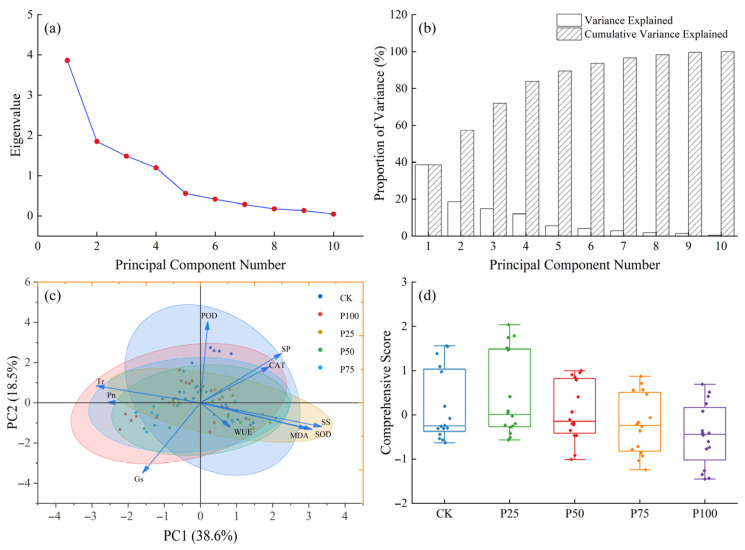
Principal component analysis of photosynthetic physiological characteristics, antioxidant enzyme activities, and soluble substances in *Caragana tibetica*. (**a**) Eigenvalue plot; (**b**) Variance explained plot; (**c**) Principal component analysis (PCA); (**d**) Comprehensive score. Ellipses represent the 95% confidence regions of sample scores for each treatment group in the PC1–PC2 space.

To reveal the intrinsic relationships among various physiological indicators of *C. tibetica* under different stubble treatments and their overall response characteristics, principal component analysis (PCA) was conducted on the measured parameters. As shown in Figure 4a, the eigenvalue of the first principal component (PC1) is significantly higher than those of the other components, while the second principal component (PC2) is also prominent; the subsequent eigenvalues decrease sharply, indicating that the first two principal components can adequately represent the major variation in the original data. The corresponding variance contribution bar chart (Figure 4b) further confirms that PC1 and PC2 together explain 57.1% of the total variance. As illustrated in Figure 4c, samples cluster according to different stubble treatments (CK, P25, P50, P75, P100), exhibiting a certain distribution trend. Overall, photosynthetic physiological indicators are negatively correlated with antioxidant characteristics. Specifically, Pn, Tr, and Gs show strong negative correlations with SS, SP, as well as SOD and CAT along the PC1 direction, indicating that these indices collectively reflect the plant’s photosynthetic efficiency and stress resistance. On the other hand, the negative correlation of Gs and WUE with POD, SP, and CAT along the PC2 direction suggests a certain degree of functional complementarity or trade-off between water regulation mechanisms and antioxidant defense. The comprehensive score box plot (Figure 4d) demonstrates differences in the integrated responses among the stubble treatments, with the P25 and P50 groups achieving relatively higher comprehensive scores, reflecting their superior overall performance compared to the control and other treatments. This indicates that appropriate stubble treatment can help improve the physiological status and stress resistance of *C. tibetica*.

In summary, PCA analysis reveals a complex network of relationships among the physiological indicators of *C. tibetica*, and shows that different stubble treatments significantly affect the plant’s overall physiological performance by regulating photosynthetic characteristics and antioxidant systems. This provides a theoretical basis for optimizing management practices and improving the growth and stress resistance of *C. tibetica*.

### 2.5. Comprehensive Evaluation of Stress Resistance in Caragana tibetica Based on Principal Component Analysis and Optimal Stubble Height Analysis Using the Generalized Additive Model

**Figure 5:**
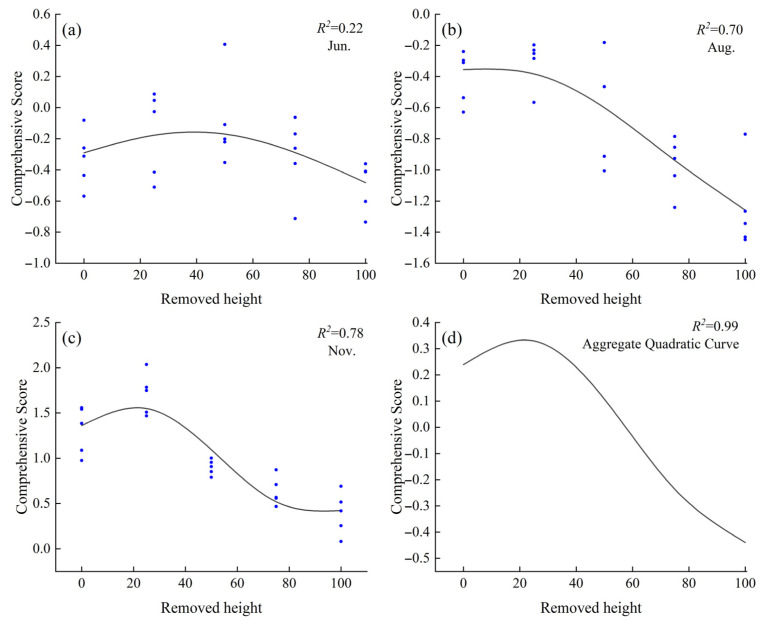
Generalized Additive Model (GAM) fitting curve of the comprehensive stress resistance score of *Caragana tibetica* under different stubble heights. (**a**) June fitting curve; (**b**) August fitting curve; (**c**) November fitting curve; (**d**) Quadratic curve fitting. In the figure, the curves in (**a**–**c**) are fitted based on PCA comprehensive scores and cutting on degree, while (**d**) is a quadratic fit derived from the curves in (**a**–**c**).

Based on the comprehensive evaluation using PCA in the previous section, it was found that among the known stubble heights, *C. tibetica* exhibited the highest stress resistance at a stubble height of 25%. However, the optimal stubble height for maximizing stress resistance remained unclear. Therefore, this study applied the Generalized Additive Model (GAM) to fit the relationship between stubble height and the comprehensive PCA-based stress resistance score across different months. As shown in Figure 5, the fitted curves for June, August, November, and the combined data reveal a trend where the comprehensive score initially increases and then decreases with rising stubble height. This suggests the existence of a theoretical optimal stubble height. In June, the proportion of variance explained (PVE) was 28.2%, with an *R*^2^ of 0.22, indicating limited explanatory power of stubble height for the comprehensive score. In August, the PVE was 72.7% and *R*^2^ was 0.70. The highest stress resistance was observed at a stubble height of 8.0%; compared to CK, stubble heights ranging from 0.5% to 14.0% improved stress resistance, with no significant difference from the CK in comprehensive scores. In November, the PVE was 80.6% and *R*^2^ was 0.78, with the highest stress resistance at a stubble height of 21.6%. Using CK as a baseline, stubble heights between 0.5% and 38.7% promoted stress resistance in *C. tibetica*. The comprehensive model constructed using the averaged data from June, August, and November exhibited a trend similar to that of November, with a PVE of 99.9% and an *R*^2^ of 0.99. The optimal stubble height was likewise 21.6%, with the optimal range matching that found in November.

## 3. Discussion

Photosynthesis is not only the foundation of plant growth and development, but also a key factor determining productivity [18]. Moreover, photosynthesis is highly sensitive to changes in the external environment and can significantly reflect the stress resistance of plants [19,20]. In addition, the phenomenon where the overall photosynthetic rate increases after the removal of apical leaves is defined as “compensatory photosynthesis.” This process refers to the enhancement in photosynthetic capacity in the remaining tissues after damage, achieved by regulating the source-sink relationship and photosynthetic metabolism, thereby maintaining the energy and material supply required for growth and repair [21,22]. Compensatory photosynthesis is closely related to stomatal conductance, as higher stomatal conductance can enhance the photosynthetic rate [23]. Research has shown that plants can enhance compensatory growth after topping, thereby increasing their biomass [24].

This study found that, one month after cutting (at the early stage of the growing season), the Pn of *Caragana tibetica* Kom. (*C. tibetica*) first increased and then decreased with increasing cutting intensity, with P25 being the highest. In the middle and late stages of the growing season, Pn showed a trend of first decreasing and then increasing, with P50, P75, and P100 gradually increasing. Meanwhile, stomatal conductance and water use efficiency of *C. tibetica* exhibited similar trends. Research has shown that when plants are subjected to external stimuli, their photosynthetic capacity increases, and WUE changes with the variation in photosynthesis [25,26]. This phenomenon may be due to the fact that, in the early stage after cutting, leaves were not fully mature under high-intensity cutting, While mature new leaves that developed after a period of growth may possess higher photosynthetic potential due to their increased chlorophyll content [27,28]. It further reflects the compensatory photosynthesis of plants. It was also found that the photosynthetic physiological characteristics of *C. tibetica* in the middle of the growing season were overall higher than those in the early and late stages of the season, indicating that plants have a higher photosynthetic capacity during peak growth periods [29]. This phenomenon may stem from the ability of plants to perform photosynthesis more efficiently and adjust stomatal conductance under favorable environmental conditions, thereby maintaining a higher carbon assimilation efficiency. After cutting, WUE in all treatments except P25 showed varying degrees of improvement compared to CK, indicating that the stress resistance of *C. tibetica* was enhanced after cutting, allowing it to survive under limited water conditions.

Malondialdehyde (MDA) is a byproduct of lipid peroxidation in plants under stress conditions, and its concentration reflects the intensity of membrane peroxidation—the higher the value, the more severe the damage. When plants are subjected to stress, soluble sugars and soluble proteins can enhance osmotic regulation, directly or indirectly scavenge reactive oxygen species (ROS) and maintain the water potential balance between plant cells and soil, thereby preserving cell turgor, and protect the structural integrity of cellular macromolecules [30]. To counteract oxidative stress caused by drought, plants have developed a complex antioxidant defense system [31]. Within the antioxidant enzyme system, superoxide dismutase (SOD) is the first to be activated in response to stress, rapidly removing O_2_^−^. Subsequently, catalase (CAT) and peroxidase (POD) activities increase to eliminate H_2_O_2_, preventing its accumulation [32]. This coordinated action enhances the overall function of the plant’s antioxidant system. Such synergy not only helps mitigate oxidative damage but also significantly improves the plant’s adaptability to adverse environmental conditions [33]. This study found that, after cutting, the contents of soluble sugars and soluble proteins in *C. tibetica* under the P25 treatment were significantly higher compared to CK, and the activity of POD increased to varying degrees at the late stage of the growing season. Research has shown that plants exhibit greater stress resistance after cutting, especially under harsh weather conditions [34]. At the same time, the MDA content in some areas treated with cutting was higher than in the CK, indicating that the degree of damage in these areas is greater than in the CK. Studies have shown that MDA not only reflects the degree of plant damage, but at low concentrations, it can act as a stress signaling molecule to regulate the upregulation of stress response genes in plants [35]. This study suggests that the *C. tibetica* is under stress, but the concentration has not exceeded its tolerance limit. At this point, MDA, as a signaling molecule, regulates the photosynthesis of *C. tibetica*. In summary, *C. tibetica* exhibits enhanced stress tolerance under extreme temperatures after being cut back, which aids its survival.

A single indicator cannot fully reflect the response capacity of *C. tibetica* to cutting treatments, nor can it be used to screen for the optimal cutting intensity. Comprehensive analysis using multiple physiological and biochemical indicators provides a more complete assessment of the stress resistance of *C. tibetica*. To further elucidate the intrinsic relationships among the physiological parameters of *C. tibetica*, this study employed correlation analysis and principal component analysis (PCA) to comprehensively evaluate various physiological parameters. The generalized additive model (GAM) was then used to fit the relationship between cutting intensity and the comprehensive PCA score in order to determine the optimal cutting intensity [36,37].

In the early growing season, photosynthetic physiological parameters showed a significant positive correlation with soluble substance content and antioxidant enzyme activity, indicating active photosynthesis and metabolic synthesis, abundant energy, and promotion of protein and sugar accumulation [38]. In the middle and late growing seasons, however, photosynthetic physiological parameters were significantly negatively correlated with soluble substance content and antioxidant enzyme activity. During these periods, plant growth gradually entered the maturity or senescence stage, while environmental stress increased. This led to restricted stomatal regulation and photosynthetic activity, decreased stomatal conductance, and enhanced antioxidant enzyme activity in response to oxidative stress [39]. In summary, the correlations among physiological indicators change over time [40].

The results of the principal component analysis showed that the P25 treatment conferred the strongest stress resistance. The generalized additive model revealed that the optimal cutting intensity varied among months, with a trend of first increasing and then decreasing with respect to stress resistance. The overall optimal cutting intensity was approximately 21.6%. Overall, this study enriches the theoretical basis of physiological ecology for *C. tibetica* and provides scientific guidance for the management of desert steppe ecosystems. Future research should further integrate molecular biological approaches to explore the regulatory networks underlying stress resistance mechanisms, thereby deepening our understanding and utilization of *C. tibetica*’s adaptability.

## 4. Materials and Methods

### 4.1. Overview of the Study Area

The field experiment was conducted in the central part of Darhan Muminggan Joint Banner, Baotou City, Inner Mongolia Autonomous Region, China (longitude 110°5′43″–110°6′42″ E, latitude 42°1′41″–42°1′7″ N). The landform belongs to the hilly grassland region on the northern slope of the Yinshan Mountains, with an average elevation of 1376 m. The area experiences a temperate, semi-arid continental climate, and the grassland type is classified as typical desert steppe. The prevailing wind directions throughout the year are north and northwest. The meteorological conditions during the experiment were generally favorable for the growth of *C. tibetica* (see Appendix A for basic meteorological data). The vegetation consists mainly of perennial xerophytic herbs adapted to arid and semi-arid temperate climate conditions, dominated by *Stipa tianschanica* var. *gobica* (Roshev.) P. C. Kuo & Y. H. Sun. The community structure of the grassland vegetation is simple, with short and sparse grass layers. The main species include *Stipa krylovii* Roshev., *Artemisia frigida* Willd., *Caragana microphylla* Lam., and *Thymus mongolicus* (Ronniger) Ronniger.

### 4.2. Experimental Design

To minimize the interference from human activities and grazing in the experimental area, the research team implemented enclosure management in April 2023 on plots within the study area where populations of *C. tibetica* are densely distributed. The purpose of the enclosure was to provide undisturbed, naturally growing experimental subjects for subsequent clipping experiments, thereby ensuring the reliability of the experimental results.

**Figure 6:**
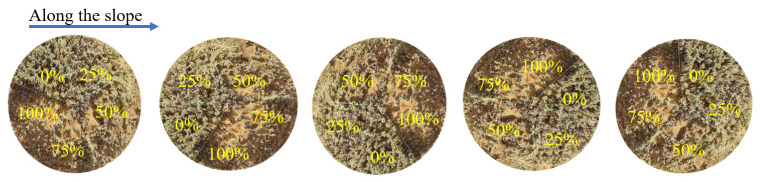
Schematic diagram of the flat stubble pattern. The percentages in the figure represent the proportion of shoots pruned from top to bottom in the area.

At the end of May 2024, a clipping experiment on *C. tibetica* was conducted within the previously enclosed plots. To eliminate the influence of slope differences on shrub growth, all samples were selected from areas with consistent slope and aspect. Within the enclosed plots, healthy *C. tibetica* shrubs with an approximately circular canopy and a diameter of about 1 m were selected along the slope direction. For each shrub, the canopy was divided into five equal sectors by taking the center of the canopy as the origin and partitioning at intervals of 72°, ensuring equal spatial area for each sector (Figure 6). The distribution of branch quantity and length within each sector was kept as balanced as possible to minimize sample variation among sectors.

The five sectors of each *C. tibetica* shrub were sequentially assigned to different clipping treatments: CK (0%), P25% (25%), P50% (50%), P75% (75%), and P100% (100%). Each clipping intensity was assigned to a separate sector on each shrub, with five shrubs used as replicates for each treatment, thus enabling a controlled experiment of different clipping intensities within individual shrubs.

### 4.3. Index Measurements

#### 4.3.1. Photosynthetic Index Measurement

In June (early growing season), August (middle growing season), and November (late growing season) of 2024, under clear weather conditions between 09:00 and 11:00 in the morning, in situ measurements of *C. tibetica* were conducted under natural light using a portable photosynthesis measurement system GFS-3000 (Heinz Walz GmbH, Effeltrich, Germany; GFS-3000 Gas-Exchange System, WALZ). Experimental materials were selected from the top, fully expanded functional leaves (the first fully expanded leaf from the top downward) of representative *C. tibetica* branches with uniform growth and different clipping intensities across the five replicates. The measured indices included: net photosynthetic rate (Pn), transpiration rate (Tr), stomatal conductance (Gs), and water use efficiency (WUE). The formula for calculating WUE is as follows:

(1) WUE=Pn/Tr

In the formula, WUE represents leaf water use efficiency; Pn is the net photosynthetic rate; and Tr is the transpiration rate.

#### 4.3.2. Measurement of Physiological Indices

The growth and physiological indicators of the newly grown leaves of *C. tibetica* after cutting were measured. Catalase (CAT) activity was determined using the UV absorption method. The *C. tibetica* samples were diluted into a suspension, and the reaction mixture was prepared by mixing the diluted sample extract, phosphate-buffered solution, KCoCl_2_, and H_2_O_2_ in a test tube. The CAT catalyzes the breakdown of H_2_O_2_, and the formation of bubbles was observed after the reaction stopped. A spectrophotometer was used to measure the absorption at 240 nm to assess CAT activity [41]. Peroxidase (POD) activity was measured using the guaiacol method. During plant sample extraction, H_2_O_2_ and PBA solution were added, and the absorption at 470 nm was measured with a spectrophotometer to evaluate POD activity [42]. Superoxide dismutase (SOD) activity was determined using the nitroblue tetrazolium (NBT) reduction method. The sample extract was reacted with NBT solution, yellow hydrogen oxidase solution, NaH_2_PO_4_ solution, and Na_2_AsO_2_ solution. After stopping the reaction, the absorbance at 560 nm was measured using a spectrophotometer, and the SOD activity was quantified using a standard curve [43]. Malondialdehyde (MDA) content was measured using the thiobarbituric acid (TBA) method. The *C. tibetica* samples were heated in phosphate-buffered solution, and the absorption at 532 nm was measured using a spectrophotometer. MDA content was calculated based on the standard curve [44]. Soluble sugar (SS) content was determined using the anthrone colorimetric method. The *C. tibetica* samples were ground into powder and placed in a 50 mL centrifuge tube. Distilled water was added, and SS was extracted using a boiling water bath. After centrifugation to remove insoluble substances, the supernatant was reacted with anthrone and the color was developed in a boiling water bath. After cooling, the final absorption at 620 nm was measured using a spectrophotometer, and the SS content in the sample was calculated based on the standard curve [45]. Soluble protein (SP) content was determined using the Coomassie Brilliant Blue method. The *C. tibetica* samples were ground and placed in a 50 mL centrifuge tube. Then, 10 mL of trichloroacetate buffer was added to homogenize the sample in an ice bath. After centrifugation to extract the supernatant, different concentrations of BSA standard and Bradford reagent were added to the standard samples and test tubes, and the reaction time was 25 min. Finally, the absorption at 595 nm was measured using a spectrophotometer, and the protein concentration in the sample was calculated based on the standard curve [46].

### 4.4. Data Analysis

Raw data were organized and pre-processed using Microsoft Excel 2019 (Microsoft, https://www.microsoft.com, Redmond, WA, USA). Photosynthetic parameters and antioxidant characteristics were subjected to homogeneity of variance testing (Levene’s test) and normality distribution testing (Kolmogorov–Smirnov test) using IBM SPSS Statistics 25.0 (IBM, https://www.ibm.com, Armonk, NY, USA), followed by one-way analysis of variance (ANOVA) to evaluate the effects of different treatments and time points on each physiological parameter. Correlation analysis was conducted using the Pearson method to explore relationships among the various indicators. Principal component analysis (PCA) and comprehensive score calculation were performed on the R 4.3.2 platform (R Core Team, https://www.r-project.org, Global open-source community), utilizing the tidyverse package for data standardization, principal component extraction, and comprehensive evaluation. Comprehensive scores of different treatment groups were visualized with box plots, and the nonlinear response trends of comprehensive scores under different treatments were analyzed using generalized additive models (GAM) with the mgcv package. All visualizations, including bar charts, correlation heatmaps, and PCA plots, were completed with Origin Pro 2024 (OriginLab, https://www.originlab.com, Northampton, MA, USA). All statistical data are presented as mean ± SD to ensure consistency. Statistical significance is indicated using standard symbols: *p* ≤ 0.05, *p* ≤ 0.01, *p* ≤ 0.001, *p* > 0.05 (ns).

## 5. Conclusions

Moderate cutting can enhance the stress resistance of *C. tibetica*, and significantly improve photosynthetic physiological parameters and the accumulation of soluble substances, as well as increase its antioxidant capacity. Specifically, after moderate cutting, *C. tibetica* triggers compensatory photosynthesis, elevating its net photosynthetic rate and water use efficiency, which enables the plant to maintain high productivity under limited water resources, accumulate greater biomass and exhibit greater stress resistance. The correlation between photosynthetic physiological parameters and antioxidant substances changes across different growth stages, showing a significant positive correlation in the early growing season, and a significant negative correlation in the middle and late growing seasons. Using a generalized additive model, the optimal cutting intensity was determined to be 21.6%. This study demonstrates that moderate cutting can improve the environmental adaptability of *C. tibetica*, providing a scientific basis for maintaining the health of desert steppes and achieving the dual goals of sustainable grassland utilization and ecological restoration.

## Figures and Tables

**Figure A1:**
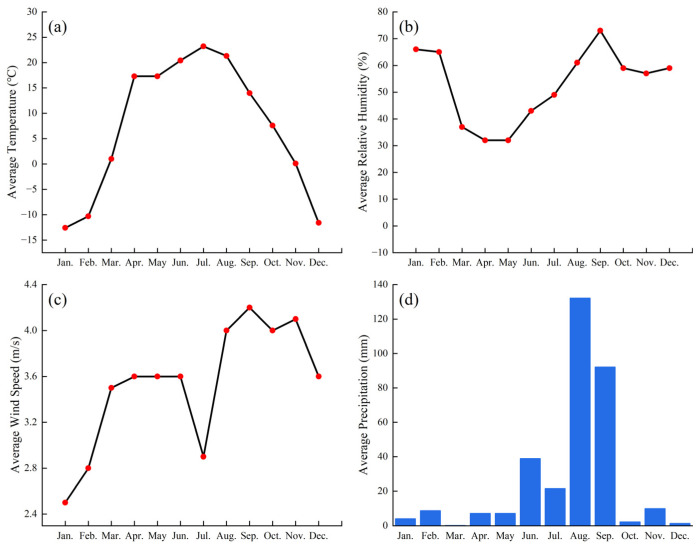
The basic meteorological conditions of the experimental field. (**a**) Average Temperature; (**b**) Average Relative Humidity; (**c**) Average Wind Speed; (**d**) Average Precipitation. The data is from the China Meteorological Data Network, website: https://data.cma.cn/.

## Data Availability

The data presented in this study are available on request from the corresponding author, since the project has not yet been completed, the data is under confidentiality.

## Appendix A. Meteorological Conditions

### Basic Meteorological Data

The annual mean temperature is 4.2 °C, with an average annual precipitation of 256 mm. The detailed data for relative humidity, annual temperature, wind speed, and precipitation are presented in Figure A1.
